# Effect of yeast probiotic *Saccharomyces boulardii* cell wall extract on *Aspergillus fumigatus* allergenicity in A549 cells

**DOI:** 10.22034/cmm.2024.345134.1463

**Published:** 2023-12

**Authors:** Donya Nikaein, Haleh Malekmadani, Babak Beikzadeh, Reza Mardanpour, Alireza Khosravi, Seyed Mohammad Moghadami

**Affiliations:** 1 Department of Microbiology and Immunology, Faculty of Veterinary Medicine, University of Tehran, Tehran, Iran; 2 Department of Cell and Molecular Biology and Microbiology, Faculty of Biological Sciences and Technology, University of Isfahan, Isfahan, Iran

**Keywords:** *Aspergillus fumigatus*, Asp f1, Cell wall extract, Fungal allergy, Interleukin 13, Interleukin 17, *Saccharomyces boulardii*

## Abstract

**Background and Purpose:**

Interest in probiotic use for respiratory allergies has increased. In this regard, the present study aimed to evaluate the effect of cell wall extract of *Saccharomyces boulardii* on *Aspergillus fumigatus* as an allergenic fungus and its effectiveness in reducing inflammatory cytokines in A549 cells sensitized with *A. fumigatus* conidia.

**Materials and Methods:**

Cell wall of *S. boulardii* was prepared and challenged by *A. fumigatus* conidia at various concentrations. Secretory protease activity was tested using the Casein method. The *A. fumigatus* allergen 1 (Asp f1) gene expression was calculated by quantitative real-time polymerase chain reaction (qRT-PCR). In another experiment, qRT-PCR was used to examine gene expression of interleukin 13 and interleukin 17 by A549 lung epithelial cells exposed to *A. fumigatus* conidia and treated with different concentrations of *S. boulardii* cell wall extract.

**Results:**

*Saccharomyces boulardii* cell wall extract significantly reduced the protease activity of *A. fumigatus* at concentrations of 10 and 20 mg/ml (*P*<0.05). The Asp f1 gene expression was significantly down-regulated in each concentration of *S. boulardii* cell wall extract (*P*<0.05). *Aspergillus fumigatus* conidia upregulated the expression of IL-13 and IL-17 in A549 cells, and *S. boulardii* cell wall extract could downregulate the expression of the mentioned cytokines at concentrations of 10 and 20 mg/ml (*P*<0.05).

**Conclusion:**

According to the results, it can be concluded that *S. boulardii* cell wall extract could be a candidate for IL-13- and IL-17-induced Aspergillus-mediated allergy and asthma therapies. Nevertheless, future studies need to be conducted on the safety of *S. boulardii* cell wall extract *in vivo* and its effects on other arms of allergic hypersensitivity.

## Introduction

Allergy is a hyperimmune reaction to an antigen called an allergen. Approximately one billion people worldwide suffer from allergies and it is estimated that this number will increase to four billion in the next 40 years [ 1 ]. Fungi are eukaryotic organisms that cause disease or act as allergens and can trigger or exacerbate respiratory diseases, including asthma [ 2 ]. *Alternaria*, *Aspergillus*, *Cladosporium*, and *Penicillium* spp. are the most common triggers of fungal allergies and some of their allergens have been identified [ 3 , 4 ]. It is estimated that between 3% and 10% of the population may be sensitized to fungi, and this rate could reach up to 50% in inner cities [ 5 ]. 

*Aspergillus fumigatus* is the most important pathogenic species of the genus *Aspergillus*. It produces conidia with the size of 2.5-3.5 µ that can invade the lower parts of the lungs after inhalation [ 6 ]. It causes allergic reactions, including allergic alveolitis, asthma, allergic sinusitis, chronic eosinophilic pneumonia, hypersensitivity pneumonitis, severe asthma with fungal sensitization, and allergic bronchopulmonary aspergillosis (ABPA) [ 7 ]. The ABPA is the most serious form of allergy to *A. fumigatus*. This condition could be associated with lung damage and asthma [ 8 ]. Studies have shown that patients with ABPA experience wheezing and shortness of breath when exposed to *A. fumigatus*, and in severe cases, this reaction can be life-threatening. *Aspergillus fumigatus* has five known major allergens (Asp f 1-4 and Asp f 6), of which Asp f 1 is the most important protein [ 9 ]. 

However, there is no effective treatment for fungal allergies, and therapies are mostly based on steroids and antihistamines, which only relieve allergic symptoms. Immunotherapy is another proposed treatment method. Nevertheless, according to the recommendations of the World Health Organization, it is difficult to achieve its effectiveness in fungal allergies and therefore, is not yet available for the treatment of fungal allergies [ 10 ]. 

Probiotics are live microorganisms that have the potential to treat and prevent a variety of diseases [ 11 ]. They are considered an alternative therapy for preventing and relieving allergic symptoms [ 12 ]. Recent research suggests that probiotics could also be used to treat and prevent respiratory diseases [ 13 ]. They promote host health through their immunomodulatory effects and activation of host defense pathways [ 1 ]. The first clinical trial on the use of probiotics to prevent allergies was conducted in 2001 and revealed that consumption of *Lactobacillus rhamnosus* during late stages of pregnancy and in newborns can reduce the prevalence of atopic eczema [ 14 ]. It is claimed that probiotics modulate both cellular (T helper [Th] 1, Th2, Th17, T regulatory [Treg] and cytotoxic T cells [CTLs]) as well as humoral (B cells) immunity [ 14 ]. Probiotics can modulate serum cytokines and immunoglobulin E (IgE) and also reduce eosinophilia in asthma patients [ 15 ].

*Saccharomyces boulardii* is a unique yeast probiotic used in the treatment of gastrointestinal diseases, especially as a complementary treatment along with antibiotics. Studies have found that its properties are independent of intestinal colonization [ 16 ]. Since there is a knowledge gap regarding the potential use of probiotics to prevent allergic diseases, the present study aimed to investigate the effect of *S. boulardii* cell wall (CW) extract on *A. fumigatus* as an allergenic fungus and determine its effectiveness in the reduction of inflammatory cytokines in sensitized A549 cells with *A. fumigatus* conidia.

## Materials and Methods

### Saccharomyces boulardii cell wall extract preparation

*Saccharomyces boulardii* (ATCC 74012) was purchased from the collection of Mycology Research Center. The CW extract was prepared based on the instructions provided by Smith et al. (2016) with some modifications [ 17 ]. Briefly, *S. boulardii* was cultured on yeast-peptone-dextrose medium (YPD) for 48 h at 30 °C and 150 rpm. Afterward, the yeast suspension was centrifuged at 2,500 g for 10 mins. The biomass of the cells was resuspended in 10 mM Tris-HCl, pH 7.5. Yeast cells were autolyzed for 24 h at 50 °C and stirring at 200 rpm. Autolyzed cells were centrifuged again at 9,000 g for 10 min in 4 °C and washed with 1 M NaCl. The CWs were extracted with 50 mM Tris-HCl detergent consisting of 2% sodium dodecyl sulfate, 100 mM ethylenediaminetetraacetic acid (EDTA), and 40 mM 2-ME 5 min at 90 °C. Yeast CWs were then washed with sterile phosphate-buffered saline and resuspended in phosphate-buffered saline (PBS). The extracts were cultured on YPD agar for 48 h at 30 °C to confirm the absence of growth [ 17 ]. The CW extracts were finally freeze-dried and stored at −20 °C until use. 

### Aspergillus fumigatus conidia suspension

*Aspergillus fumigatus* (ATCC 90906) was provided by the Mycology Research Center, University of Tehran, Tehran, Iran. It was cultured on Sabouraud dextrose agar with chloramphenicol (0.05 g/L) (SC) for 7 days at 30 °C. *Aspergillus* conidia were collected by washing the cultured fungus with PBS plus 0.05% Tween-20 solutions and centrifugation at 300 g for 10 min. Precipitated conidia were resuspended in sterile PBS and counted with a Haemocytometer. The final suspension was adjusted to 1×10^6^ cells/ml. Viability of *A. fumigatus* conidia was over 99% by culturing the conidia suspension on Sabouraud dextrose media [ 18 ]. 

### Protease assay using the Casein method

*Aspergillus fumigatus* conidia were grown at a concentration of 1×10^6^ cells/ml on 250 ml YPD medium (30 °C, 150 rpm) for five days, and *S. boulardii* CW extract was prepared at six concentrations (0-20 mg/ml, serial two-fold dilutions prepared) added to the culture media. The cultures were then centrifuged at 10,000 g, 4 °C, and 15 min. The extracellular protease activity in the supernatant of the *A. fumigatus* cultures was measured according to Oyeleke et al. (2010) [ 19 ]. 

The samples were incubated with 0.5% casein in 0.1 M Tris-HCl, pH 8, for 60 min at 40 °C. The reaction was stopped by the addition of 10% cold trichloroacetic acid to each sample. The samples were left at room temperature for an additional 30 min and then centrifuged at 8,000 g for 5 min. The absorbance was read at 540 nm with a spectrophotometer. A single enzyme activity was reported as a 0.1-fold increase in absorbance after 1 h of incubation at 40 °C [ 19 ].

### Quantification of Asp f1 gene expression in Aspergillus fumigatus

Asp f1 gene expression was measured using an *A. fumigatus* culture from a previous experiment. *Aspergillus fumigatus* cells were ground with liquid nitrogen using a mortar and pestle [ 20 ]. Total RNA was obtained using Trizol reagent (Ambion Life Technologies) according to the instructions of the manufacturer. The RNA was reverse transcribed into cDNA using the SinaClon First Strand cDNA Synthesis Kit (Cat. No.: RT5201). The primers specified in this study are summarized in Table 1.

**Table 1:** Primer sequences, amplicon size, and melting temperature of the primers in the present study

Gene	Primer (5´ to 3´)		Amplicon size (bp)	Melting temperature (°C)
Asp f1	F	ACGCTCGTGCGACCTGGACATGC	140	58
	R	GCCGTCGGAAAGAGGTGCGTG		52
Β-tubulin	F	CGACAACGAGGCTCTGTACG	170	60
	R	CAACTTGCGCAGATCAGAGTTGAG		56
IL-13	F	ACGGTCATTGCTCTCACTTGCC	158	64
	R	CTGTCAGGTTGATGCTCCATACC		65
IL-17	F	CGGACTGTGATGGTCAACCTGA	155	64
	R	GCACTTTGCCTCCCAGATCACA		64
GAPDH	F	ACACAGGCTGGTGGACAG	61	64
	R	TGTTGCAAGGCGGCATT		64

IL: interleukin, Asp f1: *Aspergillus fumigatus* allergen 1, GAPDH: Glyceraldehyde 3-phosphate dehydrogenase

The expression of the β tubulin was also tested as a housekeeping gene. Real-time polymerase chain reaction (RT-PCR) amplification was performed using a Rotor-Gene Q system (Qiagen Co., Hilden, Germany) and the SYBR-Green method. The thermal cycling condition included one cycle at 50 °C for 1 h followed by 40 cycles at 94 °C for 30 s, at 55 °C for 30 s, and at 72 °C for 60 s with a final extension at 72 °C for 8 min. The RT-PCR results were analyzed according to the ∆∆CT method (Livak).

### Measurement of A549 cell viability in proximity to Saccharomyces boulardii cell wall extract using the MTT method

The human A549 cell line was grown on 25 cm^2^ flasks in Dulbecco's Modified Eagle Medium (DMEM) with 5% fetal bovine serum (FBS) at 37 °C and 5% CO_2_. When flasks were confluent, the cells were trypsinized and cultured in 96-well plates at a concentration of 5 × 10^4^ cells per well in 100 µl DMEM medium and serial two-fold dilutions of *S. boulardii* CW extract, at 37 °C, 5% CO_2_ for 18 h. After incubation, the medium was removed and 100 µl of MTT solution (5 mg/mL) was added to each well. The cells were incubated for 3 h and then 100 µl of DMSO reagent was poured into each well to stop the reaction and dissolve formazan crystals. Optical density was read with an ELISA reader at 570 nm and a reference filter of 630 nm. Cell viability was measured using the following formula [ 21 ]. 

$\mathrm{\%Validity}=\frac{\mathrm{Mean Absorbance of Sample}}{\mathrm{Mean Absorbance of Control}}*100$

### Culture of human lung epithelial cells (A549) with Aspergillus fumigatus and Saccharomyces boulardii cell wall extract

In the present study, the method proposed by Zhang et al. (2005) was used with some modifications [ 22 ]. Briefly, human A549 cells were grown overnight on serum-free DMEM medium in 6-well plates at 37 °C and 5% CO_2_. After incubation, A549 cells were washed with serum-free DMEM once and cultured for 18 h with *S. boulardii* extract and *A. fumigatus* conidia at 37 °C and 5% CO_2_ on serum-free DMEM medium. Saccharomyces boulardii CW extract was added to the wells at concentrations of 0-20 mg/ml (in serial two-fold dilutions). *Aspergillus fumigatus* conidia was added to all wells at a concentration of 1×10^6^/ml. 

The A549 culture without *A. fumigatus* and *S. boulardii* cell extract was counted as a negative control and A549 culture with *A. fumigatus* and without *S. boulardii* CW extract was considered a positive control [ 22 ]. 

### Interleukin 13 and Interleukin 17 gene expression in A549 cells exposed to Aspergillus fumigatus conidia

After 18 h of incubation, A549 cells were harvested using 1 ml trypsin-EDTA. The DMEM with 5% FBS was used to inactivate trypsin. The cells were centrifuged at 4 °C and 300 g for 5 min. The pellet was used for RNA extraction, and total RNA was extracted using Trizol reagent (Ambion Life Technologies) according to the instructions of the manufacturer. The purity and quantity of the isolated RNA were estimated using a nanodrop. The RNA was reverse transcribed into cDNA using the SinaClon First Strand cDNA Synthesis Kit (Cat No: RT5201). 

The primers used to amplify the interleukin (IL)13 and IL-17 cDNAs are listed in Table 1. The mRNA sequence of Glyceraldehyde 3-phosphate dehydrogenase was also evaluated as a housekeeping gene. Quantitative RT-PCR (QRT-PCR) was performed on a Rotor-Gene Q system (Qiagen Co., Hilden, Germany) using the SYBR Green method. Thermal cycling conditions were one cycle at 95 °C for 10 min, followed by 40 cycles at 94 °C for 30 s, at 60 °C for 60 s, and at 72 °C for 60 s with a final extension at 72 °C for 8 min. The RT-PCR results were analyzed using the ∆∆CT method (Livak). 

### Statistical analysis

Data were analyzed in GraphPad Prism software (version 8). Analysis of variance and Tukey post hoc test were used to examine significant differences between results. A P-value of less than 0.05 was considered statistically significant (P<0.05). All experiments were performed in three replicates and data are presented as mean ± standard error of the mean. 

## Results

### Protease activity of Aspergillus fumigatus conidia

**Figure 1:**
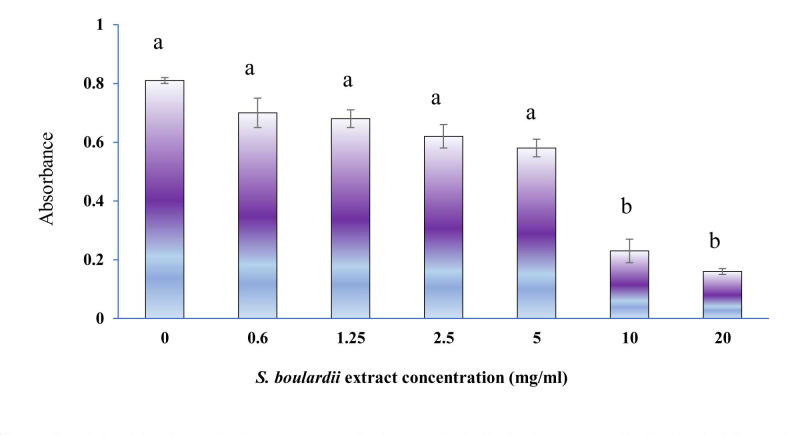
Proteolytic activity of *Aspergillus fumigatus* exposed to Saccharomyces boulardii cell wall extract assayed by Casein method. The error bar presents standard errors and different letters show significant differences (*P<0.05*). In this experiment, a *P-value* of less than 0.05 was considered statistically significant.

A decrease was observed in the protease activity of all *A. fumigatus* treated with *S. boulardii* CW extract (Figure 1). However, this reduction was significant (*P*<0.05) only at concentrations of 20 and 10 mg/mL *S. boulardii* CW extract, compared to 0 mg/ml *S. boulardii* CW extract. Therefore, the protease activity in *A. fumigatus* conidia exposed to 20 mg/ml *S. boulardii* CW extract was 15%, while this activity was 80% in the group without *S. boulardii* CW extract. No significant differences were observed between 0.6–5 mg/mL concentrations of *S. boulardii* CW extract (*P*>0.05). 

### Expression of Asp f1 gene in Aspergillus fumigatus conidia exposed to Saccharomyces boulardii cell wall extract

**Figure 2:**
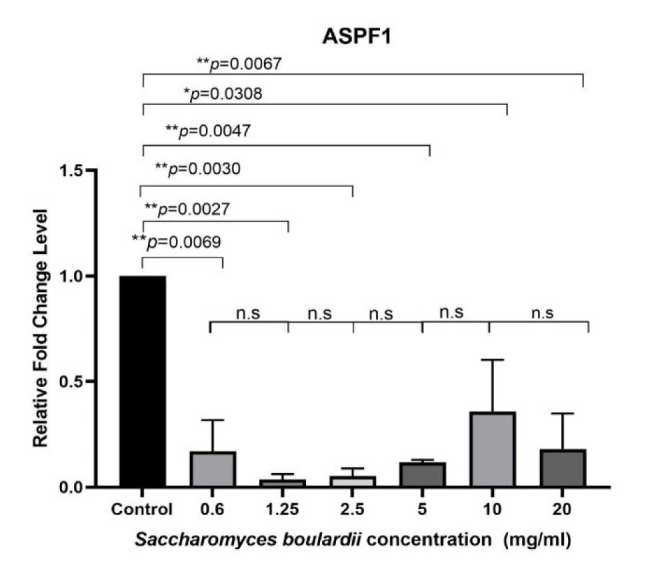
Flowchart of Asp f1 gene expression in *Aspergillus fumigatus* conidia treated with different concentrations of *Saccharomyces boulardii* cell wall extract based on relative fold change levels. Error bars show standard error. NS: non-significant; *: *P<0.05*; **: *P<0.01*. In this experiment, a *P value* of less than 0.05 was considered statistically significant.

Figure 2 shows the level of Asp f1 gene expression in *A. fumigatus* conidia treated with six concentrations of *S. boulardii* CW extract. According to the QRT-PCR results, the expression of the Asp f1 gene was significantly suppressed in all treatment groups, compared to a concentration of 0 mg/mL *S. boulardii* CW extract (*P*<0.05). Nevertheless, no significant differences were observed between concentrations of 0.6–20 mg/mL *S. boulardii* CW extract (*P*>0.05). *Saccharomyces boulardii* CW extracts at a concentration of 10 mg/mL exhibited the highest reduction in the expression of Asp f1 (*P*>0.05). 

### MTT assay

MTT assay was used to understand the effect of different concentrations of *S. boulardii* CW extract on the viability of A549 cells. For other experiments, the concentration at which > 90% viability was observed was selected. In the experience of the authors, the concentration of 20 mg/mL *S. boulardii* CW extract had 95% viability in A549 cells. Therefore, this concentration was selected as the first point in all experiments.

### Expression of Interleukin 13 gene by A549 cells infected with Aspergillus fumigatus and treated with Saccharomyces boulardii cell wall extract

**Figure 3:**
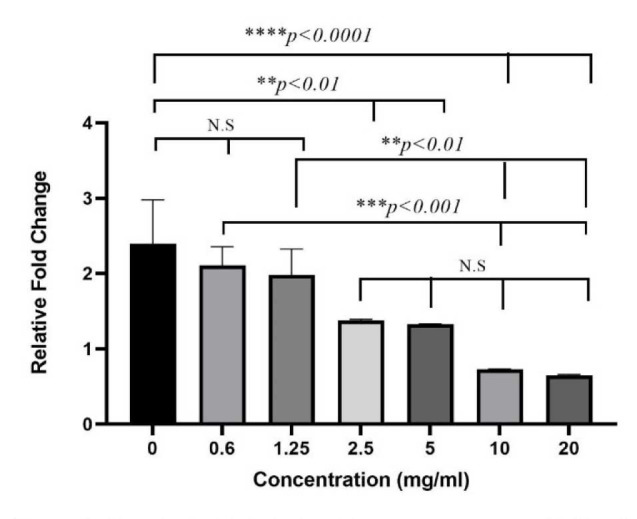
Flowchart of Interleukin 13 gene expression in A549 cell culture challenged by *Aspergillus fumigatus* conidia and treated with different concentrations of *Saccharomyces boulardii* cell wall extract based on relative fold change levels. Error bars show standard error. NS: non-significant; *: *P<0.05*; **: *P<0.01*; ***P<0.001; ****: *P<0.00001*. In this experiment, a *P value* of less than 0.05 was considered statistically significant.

Interleukin 13 gene expression was calculated according to the ∆∆CT method and the relative fold change of the treatment group was evaluated (Figure 3). *Aspergillus fumigatus* conidia upregulated the expression of the interleukin (IL) 13 gene in A549 cells. All amounts of *S. boulardii* CW extract could reduce the expression of IL-13, compared to the *A. fumigatus* group alone. However, this decrease was only significant at 2.5–20 mg/mL concentrations of the extract (*P<0.01*), while it was not significant after exposure to *A. fumigatus* conidia at the mentioned concentrations (*P>0.05*). In comparison to other treatment groups, significant differences were observed only between the concentrations 20 as wells as 10 mg/mL, with concentrations 0.6 and 1.25 mg/mL (*P<0.05*). Moreover, downregulation of IL-13 gene expression was only observed at concentrations of 20 and 10 mg/mL.

### Expression of interleukin 17 gene by A549 cells infected with Aspergillus fumigatus and treated with Saccharomyces boulardii cell wall extract

**Figure 4:**
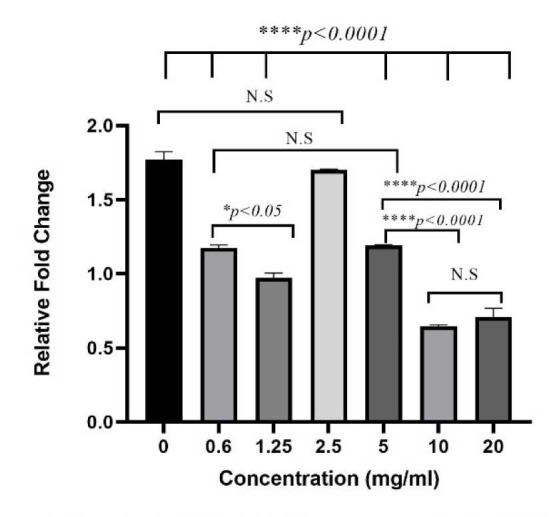
Flowchart of Interlukin17 gene expression in A549 cell culture challenged by *Aspergillus fumigatus* conidia and treated with different concentrations of *Saccharomyces boulardii* cell wall extract based on relative fold change levels. Error bars show standard error. NS: non-significant; *: *P<0.05*; **: *P<0.01*; ***P<0.001; ****: *P<0.00001*. In this experiment, a *P value* of less than 0.05 was considered statistically significant.

The IL-17 gene expression was calculated using the ∆∆CT method, and the relative fold change of different treatment groups was determined (Figure 4). 

According to the results of the present study, exposure to *A. fumigatus* could increase the expression of the IL-17 gene. Treatment with all concentrations of *S. boulardii* CW extract inhibited this up-regulation, although this was only significant at concentrations of 20 and 10 mg/mL (*P<0.05*). 

The decrease in the IL-17 gene after treatment with *S. boulardii* CW extract was highly significant in all groups, except the group with 2.5 mg/mL concentration (*P*<0.0001). The greatest decrease in IL-17 gene expression was observed at a concentration of 10 mg/mL *S. boulardii* CW extract; however, this difference in results was not significant at a concentration of 20 mg/mL. The smallest decrease in IL-17 expression was observed at a concentration of 2.5 mg/mL, and this decrease was significant in all treatment groups (*P*<0.05). 

## Discussion

Since *Aspergillus* fungi are found everywhere, exposure to this fungus can occur indoors, at home, during a hospital stay, and at work. Occurrence of the disease is highly dependent on the immune function of the host [ 23 ]. Allergic aspergillosis (rhinosinusitis and bronchopulmonary) affects approximately > 10 million people worldwide. Severe asthma is also known to be related to *Aspergillus* sensitization [ 24 ]. 

In a recent study, sensitization to *A. fumigatus* was found in 23.9% of asthma patients [ 25 ]. *Aspergillus fumigatus* is the most common Aspergillus species due to the small size of its conidia, which can penetrate into the lower respiratory tract [ 23 ]. The interaction of *A. fumigatus* candida with respiratory epithelial cells is thought to be the trigger for inflammatory and damage repair responses [ 26 ]. 

The present study examined the effect of *S. boulardii* CW extract on *A. fumigatus* secretory protease activity and Asp f1 allergen expression. The protease activity of *A. fumigatus* allergens plays an important role in the disruption of the integrity of the epithelium. Proteases are considered major allergens in fungi [ 27 ] and inhibition of proteases could prevent the production of inflammatory cytokines and protect airway epithelial cells [ 28 , 29 ]. 

In the present study, the proteolytic activity of *A. fumigatus* was 80%. A study conducted in 2012 examined the effect of EDTA and phenyl-methylsulfonyl fluoride (PMSF) as protease inhibitors in a culture of *A. fumigatus* in various substrates. The aforementioned study showed that EDTA could reduce protease activity in casein media by 10% while PMSF had no protease inhibitory activity [ 30 ]. Since EDTA inhibits metalloproteases, the authors suggested that casein is a suitable substrate for the induction of metalloproteases, while the main proteases in pig lungs were serine proteases and both proteases were inhibited in mucin. 

In the present study, by increasing the concentration of CW extract, the inhibitory effect on protease activity increased; however, a significant decrease was only observed at concentrations of 20 and 10 mg/mL. Since this research aimed to understand the effect of *S. boulardii* CW extract, yeast extract–peptone–glycerol media suitable for all proteases was used and the total protease activity was measured. In future studies, caution should be exercised when using different substrates to further identify which proteases are affected more by *S. boulardii* CW extract. 

Asp f1 is a ribotoxin expressed on the cell surface of *A. fumigatus*. It is an allergen and cytotoxin [ 31 ]. Patients with ABPA have been shown to have higher IgE levels against ASP f1 (P<0.05) [ 32 , 33 ]. Results of another investigation have shown that patients seropositive to Asp f1 and Asp f2 may be involved in *Aspergillus* bronchitis and possibly the occurrence of ABPA [ 34 ]. Therefore, down-regulation of the Asp f1 gene should prevent hypersensitivity to *A. fumigatus*. The QRT-PCR was performed to evaluate the level of ASP f1 expression as the major allergen of *A. fumigatus* upon exposure of *A. fumigatus* conidia to *S. boulardii* CW extract. In all groups treated with *S. boulardii* CW extract, the expression of Asp f1 was significantly decreased. 

In a mouse model study, the effect of intranasal truncated recombinant human SP-D (a hydrophilic protein) (rfhSP-D) was examined in mice sensitized with *A. fumigatus* culture filtrate containing Asp f1 and Asp f3. The results showed that rfhSP-D could suppress the development of allergic symptoms in hypersensitized mice [ 35 ]. Miranda et al. (2020) examined the effect of viable and supernatant cultures of *S. cerevisiae* on a mouse model of food allergy. They observed an attenuation of tissue damage and myeloperoxidase activity and a reduction in IL-17 levels, but no reduction in IgE and IgG anti-ovalbumin were obtained. They concluded that yeast had local but not systemic effects on the mouse model [ 36 ]. 

In the present study, a somewhat dose-dependent decrease in IL-17 gene expression was also observed in A549 lung epithelial cells treated with *S. boulardii* CW. However, since the extract was not studied *in vivo*, it cannot be concluded whether the results are limited to lung epithelial cells or this extract could systematically influence allergic reactions. 

In recent years, oral and nasal administration of probiotics for the prevention and treatment of allergic diseases has been discussed. However, our knowledge about the use of probiotics to prevent or treat allergic respiratory diseases is very limited. Application of wild-type and recombinant *Lactobacillus rhamnosus* GR-1 in mice to prevent allergic asthma inhibited airway hyperreactivity and the resulting deterioration in airway function [ 37 ]. A review study also concluded that probiotics are useful in allergic diseases, can modulate serum cytokines and IgE, and reduce eosinophilia [ 38 ]. In the present study, consumption of *S. boulardii* CW could reduce the expression of inflammatory cytokines IL-17 and IL-13, which is consistent with the findings of previous studies [ 36 , 38 ]. 

The IL-13 is an indicator of Th2 responses that alleviates allergic asthma symptoms, such as IgE synthesis, excessive mucus secretion, airway hyper-responsiveness, and fibrosis [ 39 ]. Rothenberg et al. (2011) showed that eosinophil recruitment and chemokine production critically depend on IL-13Rα1 (receptor for IL-13 induction) and IL-13 signaling after *Aspergillus* sensitization. In the present study, the challenge posed by A. fumigatus conidia in A549 cells regulated the expression of IL-13, showing that the importance of this cytokine in inducing allergic reactions and that only higher doses of S. boulardii CW could downregulate its expression. Other investigators have also documented the importance of IL-13/IL-4 in A. fumigatus-induced pneumonia and eosinophilia in ABPA and cystic fibrosis [ 40 - 43 ]. 

The IL-17 is a pro-inflammatory cytokine produced by Th17. The IL-17-producing Th17 cells are thought to complicate the pathogenesis of allergic diseases [ 44 ]. In allergic aspergillosis, IL-17 signaling has been documented to play a role in inflammation and tissue damage while anti-IL-17 antibodies could reduce inflammation [ 45 ]. 

Rai et al. (2018) examined the serum cytokine profile in chronic rhino-sinusitis infected with A. flavus. They found A. flavus infection in 77.5% of people and the levels of IL-1β, IL-17, IL-21, and TGFβ were significantly higher in these patients. They concluded that treatment of allergic diseases with high IL-17 levels may be challenging and the prognosis may be poor [ 46 ]. 

Our previous study examined the effect of propolis extract on cytokine production in lung epithelial cells sensitized by A. fumigatus. Propolis could decrease IL-13 and IL-17 levels in the supernatant of TC1-JHU1 cells [ 47 ]. In the present study, S. boulardii CW extract could significantly downregulate IL-17 gene expression at concentrations of 10 and 20 mg/mL. 

## Conclusion

There is very limited evidence regarding the effect of *S. boulardii* on the prevention of respiratory allergies. In this experiment, the CW of this probiotic yeast was extracted and its effectiveness was examined in modulating the allergenicity and hypersensitivity response of A549 to *A. fumigatus*. The results showed that *S. boulardii* CW extract could be a candidate for regulating IL-13- and IL-17-induced *Aspergillus fumigatus*-mediated allergy and asthma. Nevertheless, future studies need to be conducted on the safety of *S. boulardii* CW extract *in vivo* and its effects on other arms of allergic hypersensitivity. 
